# Short- and long-term cognitive and electrophysiological effects of a brief working memory training in older adults: a pilot study

**DOI:** 10.1186/s12877-025-06507-2

**Published:** 2025-11-07

**Authors:** Erika Borella, Elena Carbone, Chiara Spironelli

**Affiliations:** 1 Department of General Psychology, University of Padova, via Venezia, 8, Padova, 35131 Italy; 2 Padova Neuroscience Center, University of Padova, Padova, Italy

**Keywords:** Working memory, Cognitive training, ERP training changes, Older adults, Transfer effects, Long-term effects, Cognitive benefits, Cortical plasticity

## Abstract

**Background:**

Working memory (WM) training in aging promotes an individual’s more flexible use of their resources. Conversely, WM training neural correlates have been rarely examined. In this pilot study, we aimed to assess both behavioral and neural correlates of WM training of trained and untrained (transfer effects) tasks in the short and long term.

**Methods:**

With a double-blind, repeated-measures experimental design, 30 community-dwelling older adults (aged from 64 to 75) were randomly assigned to a training group (TG) or an active control group.

**Results:**

For the trained task, behavioral data indicated an improved WM performance, and electrophysiological data showed a lateralized event-related potential activation after training and 6 months later (follow-up) in the TG only. Clear transfer effects (*n*-back task) maintained at the follow-up appeared only on the electrophysiological level.

**Conclusions:**

These results suggest that WM training can represent a promising approach to sustain older adults’ cognitive functioning and to modulate cortical plasticity, inducing long-lasting left-lateralized activation.

**Supplementary Information:**

The online version contains supplementary material available at 10.1186/s12877-025-06507-2.

## Introduction

The role of working memory (WM), i.e., the ability to retain and manipulate information simultaneously to use in complex cognitive tasks, in explaining age-related and individual differences in cognitive functioning across the lifespan is well-acknowledged [1, 2]. WM associations and involvement in cognitive mechanisms and processes, including everyday life abilities, is unquestioned. WM linear decline with aging [3] and its related cortical and functional brain alterations [4] are also well documented. In particular, age-related changes in WM performance are known as the expression of age-related structural (particularly within the prefrontal cortex), functional and neuromodulatory changes [5].

Accordingly, a thriving interest and plethora of aging studies have been carried out aimed at developing WM training procedures to enhance WM and the associated cognitive mechanisms, thereby supporting cognitive and possibly everyday functioning. Through repeated practice and without strategy teaching, these training programs seek to improve the way older adults process information, thus promoting a more flexible use of an individual’s resources [6, 7]. Thus, WM training can improve WM-trained tasks (i.e., training gains), as well as benefits in untrained cognitive skills (i.e., transfer effects) that share and rely on similar processes or neural substrates [8, 9].

Past meta-analyses summarizing evidence from training studies involving -typically-aging- older adult samples found large and long-lasting training gains in WM tasks closely similar to those trained [7, 10, 11], but less conclusive evidence appeared concerning improvements in transfer outcomes. Transfer effects are usually weaker than training gains are, as well as variable and less enduring [7, 10, 11]. Notably, to date, few studies have examined the maintenance of WM training benefits among the older adult population [7, 10, 11], and WM training efficacy is still the focus of debate, especially when considering transfer effects and long-term benefits.

Recent reviews [12–14] suggested the study of neural correlates/changes underlying training gains and transfer effects WM training induces were important to provide a better understanding of the shared neural mechanisms and ultimately, of the training efficacy. Despite the well-known association between age-related brain changes and performance in cognitive tasks involving WM, as well as the evidence of a redistribution or reorganization of cortical networks in aging [5, 15], very few studies have analyzed neural correlates of WM training among the older adult population to date [16], with heterogeneous results.

For example, Dahlin et al. [17] found behavioral WM training gains that were associated with brain activity *increase* in the left striatum after training completion for older adults of the trained group compared with controls, without transfer effects. In Brehmer et al. [18], as well as in a more recent study [19], behavioral training gains and some transfer effects were found, together with a blood-oxygen-level-dependent imaging (BOLD) *decrease* in WM-associated regions more apparent among older trained participants compared with controls. Pergher et al. [20] WM training produced training gains and transfer effects to untrained fluid intelligence and attention measures, and older trained participants showed an *increased* P300 amplitude in parietal electrodes across training sessions. Finally, Spironelli and Borella [21] found short- and long-term improvements in a WM task similar to that trained for older trained participants compared with controls. The analysis of resting-state EEG activity showed significantly *greater* activation in left frontal electrodes in the trained group after training, with frontal oscillatory responses being correlated with better behavioral performance soon after the training as well as at the six-month follow-up assessment.

This evidence suggests that, also in aging, a WM training induces significant benefits at the behavioral level, probably depending on cortical plastic reorganization. However, the presence of methodological limitations (e.g., the lack of an active control group, included only in [18, 21]), the brain functioning assessment limited to WM outcomes (but not to transfer measures), and the lack of an examination of WM training behavioral and neural correlates in the longer term (done only in [21]) pinpoint that, among older adults, further fine-grained and complete investigations of WM training benefits at both the behavioral and the neural level are essential.

With this in mind, the present pilot study examined: (i) behavioral benefits of a WM training for typically aging older adults, and, as a novelty, (ii) psychophysiological changes due to the WM training considering not only training gains but also transfer effects – an aspect that, despite its informative role, is still missing in the aging literature. Those effects were assessed at the short- term and also at the long-term, which, although rarely done, are very informative in terms of maintenance effects of the training benefits.

We chose the Borella et al. [22] WM training procedure because it has proved to foster, at the behavioral level, not only short-term but also long-term training gains and transfer effects in various studies involving older adults [23], also in different cultural contexts [24]. One of the main characteristics of such a training is that, thanks to its procedure, it facilitates learning and promotes transfer effects by engaging multiple processes (i.e. encoding, maintaining and inhibiting information, simultaneously managing two tasks, sustaining and shifting attention). Its adaptive procedure combined with a systematic variation of the demands of the task, makes then the training constantly novel and challenging, keeping participants interested and motivated during the proposed activities. Furthermore, its brief, intensive and scheduled nature gives participants enough time to consolidate the skills they acquire, while also reducing the risk of their losing any beneficial effects of having practiced with the task. There is, then, also preliminary evidence in favor of this protocol to promote adults’ functional cortical plasticity (in terms of resting-state frontal oscillatory EEG activity) both in the short and long term [21].

Training gains were assessed using a verbal WM task closely similar to those trained, i.e., the categorization working memory span task (CWMS [25]). Transfer effects were ascertained with a WM updating task, the *n*-back task [26–29], which is thought also to involve WM -updating- processes [30].

A different transfer condition was also used to ascertain – at the electrophysiological level only – whether the WM training would promote benefits in a task where both WM and semantic skills (i.e., written language -reading- comprehension) are involved: the sentence-reading task [31]. This passive reading task can be considered a WM task if we assume that the brain automatically completes a sentence with only one semantically appropriate final word: when a semantically inappropriate word appears, a typical ERP component (i.e., the N400) is generated [31]. A prior analysis on CWMS task [25] showed that a better CWMS performance was associated with greater word recognition potential (RP) on posterior left region of interest (ROI). The sentence-reading task allowed us to test whether the WM training was able to strengthen the whole linguistic network, not only the automatic process associated with word reading and early processing.

To examine the WM training benefits, these tasks were adapted to a visual modality presentation to allow the event-related potential (ERP) data collection.

An active control group, as recommended [32], with participants involved in alternative activities and exposed to the experimenter as the TG group, was also enrolled to control for test-retest effects but also any other potential confounding effects from the TG (e.g., time spent on a task or contact with the experimenter) [32].

In line with previous evidence [7, 10, 23], at the behavioral level, we expected the WM training, compared with an active control condition, to prompt both short- and long-term training gains in the task closely similar to the ones used in the training (CWMS task). We also expected WM training to promote short- and long-term improvements in the untrained WM measure (the *n*-back task) because it involves partially common WM processes the procedure trains [21, 25, 30].

At the psychophysiological level, in a previous study [25] we investigated the ERP correlates of the CWMS task: among the automatic (i.e., N1, P1 RP) and late positive components (i.e., P200 and P300), the performance in the CWMS task was correlated with word RP only. This is an automatic ERP component sensitive to automatic skilled reading that represents the earliest recognition of linguistic materials allowing to distinguish between word-like stimuli and other visually categorized stimuli. The maximum amplitude typically appeared in parietal and parieto-temporal electrodes (thus representing the ROIs for this kind ERP component), about 200–230 ms after target stimulus onset. Therefore, in agreement with [25] we expected no between-group differences in the word RP elicited by the first word of each list (used as a control condition). We also expected the target word (i.e., the last word of each list of the CWMS task) to elicit a significantly greater word RP in temporo-parietal ROIs in the training group (TG) but not in the active control group (ACG), in particular in TG participants’ left vs. right hemisphere [25], thus revealing the WM effect at early phases of word processing. Furthermore, if the previous hypotheses were confirmed, and in line with past preliminary results [21], we also expected that this left-lateralized word RP would persist at the follow-up session, carried out 6 months after the WM training ended.

Regarding transfer effects, we expected a significant effect on the P300 component elicited by target stimuli in the *2*-back task (in TG participants only) both at short- and long-term assessments, because this task requires WM processes, which are in part overlapping with those involved in the CWMS task, also at the neural level [25]. With respect to the sentence-reading task, we expected a significant modulation of the N400 amplitude [31], in particular in the TG participants’ left hemisphere, in agreement with the greater left activation hypothesized for the CWMS task.

## Methods

### Participants

Thirty older adults (age range: 64–75 years) participated in the study. They were recruited by word of mouth and at social clubs for older people in the Northern Italy. Our inclusion criteria were as follows: (i) aged between 60 and 75 years; (ii) Italian as the mother tongue; (iii) be right-handed, ascertainable with the Edinburgh Handedness Inventory [33]; (iv) a good cognitive functioning score of 27 or more on the Mini-Mental State Examination (MMSE) [34, 35], (v) good physical and mental health, as assessed via a semi-structured interview (Health Interview [36]) and normal or corrected-to-normal vision (both self-reported and directly ascertained by asking participants to read paper versions of the stimuli resembling those used in the computerized tasks in terms of size and font; all in all, none of the participants reported visual issues during the completion of the computerized tasks).

All participants performed above the critical cut-off for their age and education in the Vocabulary test taken from WAIS-IV [37]. None of the participants reported signs of depression, as assessed with the Geriatric Depression Scale [38].

Participants were randomly assigned to two groups: the training group (TG, *n* = 15), which was involved in the WM training, and the active control group (ACG, *n* = 15), involved in alternative activities^1^.

Table 1 shows the descriptive statistics of the socio-demographic characteristics and screening measures by group. The two groups did not differ in terms of age, education, MMSE, Vocabulary and GDS scores as well as gender distribution.

**Table 1:** Means (*M*) and standard deviations (*SD*) of the socio-demographic characteristics, mood and cognitive functioning measures at baseline by group (Trained group vs. Active control one)

	Trained Group (TG) (*N* = 15)		Active Control Group (ACG) (*N* = 15)		Groups differences	
	*M*	*SD*	*M*	*SD*	*t*_*(1,28)*_	*p*
Age (years)	69.40	2.89	69.33	3.29	0.06	0.95
Education (years)	11.27	2.19	11.07	2.63	0.23	0.82
Gender:						
Males, n (%)	4 (27%)		4 (27%)			
Females, n (%)	11 (73%)		11 (73%)			
Mini-Mental State Examination	27.59	0.97	28.13	1.13	−1.46	0.17
Vocabulary	52.13	9.64	52.67	10.69	−1.44	0.89
Geriatric Depression Scale	1.73	1.28	1.93	1.79	−0.35	0.73

### Study design

**Fig. 1:**
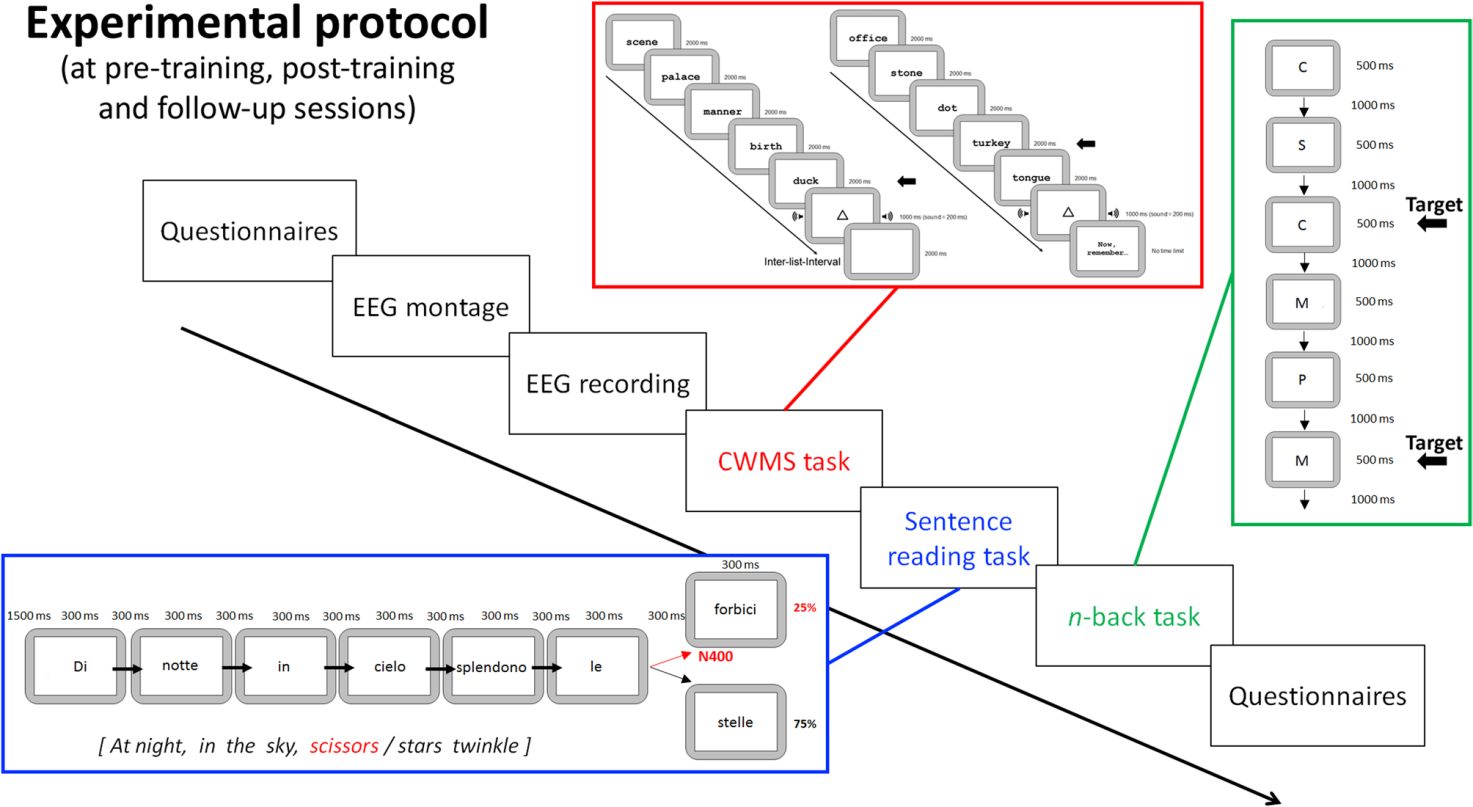
Schematic representation of the experimental protocol administered before and after the WM training (T1 and T2, respectively) as well as at the 6-month follow-up session (T3). Red box: an example of the categorization working memory span (CWMS) task. Blue box: an example of the sentence-reading task, with matching and mismatching condition (black and red colors, respectively). Green box: an example of the *2*-back task. Black arrows represent the critical item that participants must remember and refer to/recognize

This pilot study was conducted using a double-blind design. Two trained experimenters, who were not aware of the participants’ allocation in the TG or ACG, conducted the assessment sessions and the EEG collection. The training/alternative activity sessions were yielded to the TG and the ACG, respectively, by a third experimenter, who was trained in managing the two protocols, but without being aware of the aims of the study and thus of the aims of the two protocols (see the procedure section for details). Figure 1 depicts an overview of the experimental protocols.

The data collection, both behavioral and electrophysiological, took place before the training/alternative activities (T1), soon after the end of the training/alternative activities (T2) and 6 months after T2, in a follow-up session (T3). All participants were told about the aims of the study at the very end of the data collection (at follow-up). For ethical reasons, participants who were assigned to the control condition were offered to undergo the training program.

### Stimuli and tasks

Participants sat in a dimly lit room at a distance of about 60 cm from a 17-inch (screen resolution 1024 × 768) computer monitor and carried out the CWMS task, the Sentence Reading task and the *2*-back task, all programmed and administered using E-Prime software (Psychology Software Tools, Pennsylvania, USA, http://www.pstnet.com).

#### Criterion task

*Categorization Working Memory Span task* (CWMS [3]). In this computerized version of the CWMS [25], the materials consisted of 10 sets of words lists, which included 40 lists of 5 words, divided into groups containing from 2 to 6 lists of words (2 sets for each length), and displayed sequentially for 2,000 ms in the center of the white screen. After the fifth word of each list of a given set, a visual triangle (shown for 1000 ms) together with a 1000 Hz sound (presented for 200 ms) was presented to signal the end of the list. The next list from the same set was then presented 2000 ms after the triangle/sound of the previous one. Participants were required to silently read each word, visually presented, appearing in the center of the computer screen and to press the spacebar whenever an animal noun appeared (processing phase). At the end of each set, when the statement “now remember” appeared on the screen, they had to recall the last word on each list (maintenance phase) – that is, they needed to remember from 2 to 6 words, depending on the length of the set (see the red box in Fig. 1). Two computer-based practice trials containing 2 words to remember were provided before the experiment started. The experimenter wrote down recalled words on a dedicated form. Two parallel versions, such as those used in the training studies, were created, each one including 5 sets of word lists (one set for each length), which were counterbalanced across participants (further details in [25]). The total number of correctly recalled words was used as the measure of WM performance (maximum score = 20).

#### Transfer effects

*2-back task* [26–29]. The *n*-back is a sequential letter memory task that varies the level of the WM load and, therefore, the task difficulty, while keeping overall task procedures constant across conditions. We adopted the *2*-back version of the task: stimuli consisted in single consonants of the Italian language, presented – in a pseudorandom sequence – one at a time for 500 ms, in the center of the screen with an inter-stimulus-interval of 1000 ms. Participants had to press the space bar with their left hand only when the target (i.e., any letter that was identical to the one presented two trials back) appeared on the screen (see the green box in Fig. 1). All consonants of the Italian alphabet were used (i.e., no K, J, W, X, and Y letters were used) ranging between a minimum of 5 to a maximum of 12 times (depending on the type of condition). Two parallel lists were used, to counterbalance the Target/Non-target associations. There were 40 target and 80 non-target stimuli. Participants had a practice block with the *1*-back condition of the task, until they demonstrated that they understood the task rationale as well as to ensure that they had no visual issues in perceiving and reading the stimuli. The dependent variables considered were: (i) response times (RTs), (ii) accuracy rates, i.e., responses to target stimuli and non-responses to Non-target stimuli, and (iii) false alarms, i.e., responses to Non-target stimuli.

*Sentence-Reading task* [31]. In this task, stimuli consisted of 200 five-word sentences presented in 10 experimental blocks of 20 sentences each. The sentences were displayed one word at a time (this method is required when evoked potentials are recorded), with each word appearing in the center of the screen for 300 ms. The inter-word interval was 300 ms, and the inter-trial interval was 1500 ms. Participants have to read silently to answer questions about the contents of the sentences randomly between one block and the other. A random 25% of the sentences ended in a semantically inappropriate (but syntactically correct) word (see the blue box in Fig. 1).

### Electrophysiological recordings

Electrophysiological activity was recorded with 38 EEG locations, 31 tin electrodes mounted on an elastic cap (ElectroCap) according to the International 10 − 5 system [39], and the 7 more electrodes applied below each eye (Io1, Io2) on the 2 external canthi (F9, F10), nasion (Nz) and mastoids (M1, M2). All cortical sites were online referred to Cz. Data were stored with Acquire NeuroScan software, version 4.1 (Compumedics Neuroscan, Charlotte, NC, USA). Amplitude resolution was 0.1µV; bandwidth ranged from DC to 100 Hz (6dB/octave). The sampling rate was set at 500 Hz, and impedance was kept below 5 KΩ (further details in [25]).

Data were re-referenced offline to the mean activity of the whole scalp by the average reference procedure, and epoched into 2-s intervals, including 0.5 s before and 1.5 s after the onset of the stimulus of interest (i.e., the last word of each list and sentence in the CMWS and sentence-reading task, respectively, and the *2*-back letter in the *n*-back task) onset. As a CMWS control condition, we also considered the event-related activity the first word (w1) of each list elicited, using the same 2-s interval chosen for the last word of each list (further details in [25]). A 100-ms baseline preceding each stimulus was subtracted from the whole trial epoch. Single trials were corrected for eye movement artifacts (i.e., vertical and horizontal movements) and blinking. For this, BESA (Brain Electrical Source Analysis, 5.1 version) software was used to compute ocular correction coefficients [40, 41]. Each trial was then visually inspected for any residual artifacts by an expert psychophysiologist (C.S.), focusing on the critical temporal intervals for each task (i.e., 0–400 ms for the CWMS task, and 0–600 ms for both the *2*-back and the sentence-reading tasks). This *manual* procedure was preferred depending on a limited number of trials and participants, to ensure that all data were good for averaging (an automatized procedure, based on threshold criteria, is a *blind* procedure, with no control on the real quality of epochs included in the final averaging file). Overall, 26.49% of w1 trials and 26.50% of target stimuli were rejected in the CWMS task—in any case, all participants had a minimum of 15 good trials out of 24 available (further details in [25])^2^; 24.31% in the *2*-back task and 22.83% in the sentence-reading task, equally distributed among group (TG and ACG), session (pre-training, post-training and follow-up) and condition (Target/Non-target stimuli and Congruent/Incongruent stimuli for *2-*back and sentence-reading task, respectively) (disaggregated percentages and statistical analyses are available in Supplementary Materials – Part I). All accepted trials were averaged for each participant, condition, and task, regardless of their later behavioral performance (if any).

### Procedure

All participants attended six individual sessions: the first and fifth were for the pre- and post-tests, and the sixth was the 6-month follow-up (see Table 2).

**Table 2:** Description of the procedure and activities for the trained group and the active control one

Type of session and duration	Activities for Trained Group^	Activities for the Active Control Group^
1 (pre-test; 90 min.)	Edinburgh Handedness Inventory^; Health Interview^; MMSE^; GDS^; Vocabulary test^; CWMS*; Sentence Reading*; *n*-back*	
Training activities	Participants listened to sets of 2, 3, 4 or 5 word lists (each list containing 5 words)	
2 (training; 30–40 min.)	Participants were presented with three sets of word lists for each level of difficulty and were asked to remember target words (first or last one) and to tap their hand on the table whenever they heard an animal noun. This session was divided into three phases. In the first one they had to recall the first word on each list, in the second one the last word and in the final one the first word. The procedure was adaptive: If they recalled the words correctly for two of the three sets, the task’s difficulty was increased (up to sets of 5 word lists). When they were unable to do so, they moved on to the next phase of the task, which started from the easiest level (sets of 2 word lists).	Participants filled in the Autobiographic Memory Questionnaire [36]
3 (training; 30–40 min.)	Participants were presented with four sets of word lists for each level of difficulty and were asked to remember target words followed by a sound (which could be anywhere on the list) in the right order and to tap their hand on the table whenever they heard an animal noun. Animal nouns frequency also varied (i.e. from 2 to 17 animal nouns -for sets of 5 lists-). Participants had to complete the whole task, from the easiest to the hardest level of difficulty, regardless of their performance.	Participants filled in the Memory Sensitivity Questionnaire [36]
4 (training; 30–40 min.)	Participants were presented with four sets of word lists for each level of difficulty and were asked to remember target words (first or last one) and to tap their hand on the table whenever they heard an animal noun. They were asked to recall (i) the last word on each list in the first set; (ii) the first word on each list in the second set; (iii) the last word on each list in the third set; and (iv) the first word on each list in the fourth set. In this session, participants were required to complete the whole task, from the easiest to the hardest level of difficulty, regardless of their performance.	Participants filled in the Psychological Well-Being Questionnaire (BEN-SSC; [36])
5 (post-test; 60 min.)	CWMS*; Sentence Reading*; *n*-back*	
6 (3-month follow-up; 60 min.)	CWMS*; Sentence Reading*; n-back*	

*MMSE *Mini-Mental State Examination,* GDS *Geriatric Depression Scale,* CWMS *Categorization Working Memory Span task

* Tasks with ERP recording

^paper and pencil

During these three assessment sessions, each lasting about 90 min (pre-test) and 60 min (post-test and follow-up), participants completed the battery of tasks listed in Table 2. The CWMS, the *n*-back, and sentence-reading task were presented via computer to allow EEG collection, while Edinburgh Handedness Inventory, Health Interview, MMSE, Vocabulary and GDS were all paper-and-pencil tests administered at pre-test only (see Table 2 for task presentation order).

During the other three sessions (2–4), lasting about 30–40 min each and completed within a 2-week timeframe with a fixed 2-day break between each session, the TG received the WM training, whereas the ACG was occupied in alternative activities. The schedule was thus identical for the two groups, ensuring a matching amount of interaction with the experimenter.

For the trained group, the experimenter presented lists of words, audio-recorded, and organized in the same way as for the CWMS task. As for the CWMS task, the basic instructions were to recall target words and tap on the table with their hand when they heard the name of an animal (see Table 2 for details). However, some manipulations were made during the three sessions to favor generalized transfer. The maintenance demands of the training tasks were manipulated by increasing the number of words to be recalled in the case of success, and presenting the lowest memory load in the case of failure (Session 2). Moreover, the task requirements varied, requiring the recall of: (i) the last or first word of each series (Sessions 2 and 4); and (ii) words that were followed by a ‘beep’ sound (Session 3). The processing request, i.e., tapping the table when an animal noun was heard, was also manipulated by varying the frequency of these animal words in the lists (Session 3).

Participants in the ACG were required, instead, to complete questionnaires on aspects of memory and psychological well-being. In particular, they completed the Autobiographic Memory questionnaire [36], which involves recalling common events of their childhood, adulthood and recent years, and rating their vividness, in session 2. Then, in session 3, the Memory Sensitivity questionnaire was administered [36]: participants were asked to rate the frequency of behavior dedicated to saving memories of life events. In session 4, participants answered the Psychological Well-being questionnaire [36] to rate their personal satisfaction with life (past, present, and future), their emotional competence (ability to understand their own and others’ emotions), and their coping strategies regarding everyday problems.

### Statistical analyses

Preliminarily, the presence of outlying values was checked, considering as outliers behavioral scores greater or lower than 3 standard deviations (SDs) from the group average. In addition, the Kolmogorov–Smirnov test was applied to ensure that both behavioral data and every ERP component were normally distributed.

*Behavioral analyses.* To assess any group differences at the baseline (pre-test) separate *t*-test were carried out with Group as an independent variable factor (two levels: TG vs. ACG) on the pre-test performance on all tasks (except for the sentence-reading one). Results are summarized in Table S4 (Supplementary Materials – Part III).

Training effects on the measures of interest were analyzed with a between-subject ANOVA on the differences between: (i) the pre- and post-test (T2 – T1) that indicates the immediate gain index and (ii) the pre-test and the follow-up (T3 – T1) that represents the maintenance gain index. We decided to calculate gains scores instead of considering the three assessment occasions for sake of coherence across behavioral and EEG analyses (see below for further details). The ANOVA carried out on behavioral data thus included the between-subjects factor Group (two levels: TG vs. ACG) and the within-subject factor Gain (two levels: Immediate vs. Maintenance training gain index). As we had an a priori hypothesis specific of the TG, whether a significant effect involving the Group factor – but not the Gain factor – was found, the main effect was tested separately in the two samples in each gain conditions (immediate and maintenance) by means of planned comparisons. These further analyses allowed us to verify whether the effect found underwent significant changes between the pre- and post-training assessments, the pre-training and follow-up, or both.

Finally, to ascertain and -descriptively- compare the dimension of the immediate and maintenance benefits for the TG and the ACG, effect sizes (Cohen’s *d*) for immediate (post-test – pre-test) and maintenance (follow-up – pre-test) comparisons for each measure of interest were computed separately for the TG and the ACG. Values were corrected using the Hedges and Olkin [42] correction factor to avoid the small sample bias.

*ERP analyses.* Mean values of the potential measured across all participants were used for statistical analysis. In agreement with previous studies on RP [25, 43–47] and the N400 component [31, 48–51], electrodes were clustered into regions of interest (ROIs) to perform statistics.

Furthermore, as done for behavioral data, to decrease the number of statistical variables and to increase the statistical power simultaneously, an immediate gain index was computed as the difference of each ERP mean activity of post- (T2 session) minus pre-training (T1 session) on left and right ROIs, and a maintenance gain index as the difference of each ERP mean activity of follow-up (T3 session) minus pre-training (T1 session) (see Supplementary Materials – Part II for the grand-mean waveforms of electrodes selected for statistical analyses, for each outcome of interest, by group and conditions).

As the preliminary analyses carried out on pre-training (T1 session) showed no a priori differences between groups (see Tables S5-S8 in Supplementary Materials – Part III), we preferred including in the ANOVAs carried out also on EEG data the within-subject factor Gain (two levels: Immediate vs. Maintenance training gain index), rather than considering the three EEG recording sessions (i.e., T1 vs. T2 vs. T3 sessions), to lower the number of contrasts. A second advantage associated with the use of differential scores consists in the fact that they offer greater “control” of individual differences. In general, a sample of older adults (as for developmental samples) show greater variability of EEG activity [51]: the differential scores, computed within subjects, allowed us to focus on *relatively* increased or decreased cortical activation, regardless of the individual *absolute* value of participants’ EEG. Because both the RP and the N400 were negative ERP components, the training gain indices were negative when participants had higher RP or N400 amplitude after the training (immediate or maintained at follow-up), and positive when they had a higher RP or N400 amplitude before the training.

In agreement with Spironelli et al. [25], CWMS statistical analyses focused on the word RP only, centered on the RP peak, 200–230 ms for all target stimuli (last word of each list). Furthermore, as a control condition, we decided to consider the event-related activity elicited by the first word (w1) of each list: this latter represents a complementary analysis, which allowed us to verify whether only the target stimuli induced changes at cortical level. Notably, the analysis of w1 stimuli is not part of the experimental protocol and is carried out as separate from the target stimulus analysis (in agreement with our prior validation study [25]). In both these analyses, each ROI comprised 4 electrodes: Posterior Left (PL: CP3, P3, P7, TP7) and Posterior Right (PR: CP4, P4, P8, TP8). ANOVAs included the between-subjects factor Group (two levels: TG vs. ACG) and two within-subject factors: Gain (two levels: Immediate vs. Maintenance training gain index) and Laterality (two levels: Left vs. Right hemisphere).

In the *2-*back task, the statistical analysis focused on a late P300 component, 450–500 ms after stimulus onset, on two posterior ROIs of 3 electrodes each: Posterior Left (PL: P3, P7, TP7) and Posterior Right (PR: P4, P8, TP8). ANOVA was conducted including the between-subjects factor Group (two levels: TG vs. ACG) and 3 within-subjects factors: Gain (two levels: Immediate vs. Maintenance training gain index), Stimulus (two levels: Target vs. Non-target) and Laterality (two levels: Left vs. Right hemisphere).

In the *sentence-reading* task, ANOVA was conducted focusing on the N400 component, 450–550 ms after stimulus onset, calculated as the difference between mismatch and match conditions: the greater the negativity, the greater the mismatching effect. Statistical analyses were focused on two frontal and two central ROIs of 2 electrodes each: Frontal Left (FL: F3, F7), Frontal Right (FR: F4, F8), Central Left (CL: C3, CP3), and Central Right (CR: C4, CP4), including the between-subjects factor Group (two levels: TG vs. ACG) and 3 within-subjects factors: Gain (two levels: Immediate vs. Maintenance training gain index), Region (two levels: Frontal vs. Central), and Laterality (two levels: Left vs. Right hemisphere).

Post-hoc comparisons were computed using the Newman–Keuls test (*p* <.05).

Notably, in ERP data analyses we included the within-subjects Gain factor (Immediate vs. Maintenance training gain index) to limit the number of ANOVAs carried out on different conditions or stimuli in the analyses: as we had an a priori hypothesis specific of the TG, when a significant interaction involving the Group factor – but not the Gain factor – was found, this two-way interaction was tested separately in the two samples and gain conditions (immediate and maintenance) by means of planned comparisons. These further analyses allowed us to verify whether the effect(s) found underwent significant changes between the pre- and post-training assessments, the pre-training and follow-up, or both.

## Results

Results from the Kolmogorov–Smirnov test confirmed that both behavioral data (immediate and maintained gains, see below) and every ERP component were normally distributed (all *d*s ≤ 0.192, all *p*s > 0.20).

As seen in Tables S4-S8 (Supplementary Materials – Part III), results from preliminary statistical analyses revealed that there were no significant baseline differences between the TG and the ACG in both behavioral (Table S4) as well as electrophysiological data (Tables S5-S8).

### Behavioral data

#### Training gains

As seen in Table 3, results from ANOVAs showed that the two groups differ in the criterion task (CWMS), in favor of the TG, regardless of their gain indices (Group main effect: *F*(1,28) = 4.92, *p* =.03, *η*^2^_p_ = 0.15). To test the reliability of results in both immediate and maintained training gain indices, planned comparisons were carried out between groups (TG vs. ACG), revealing that the TG benefit was significant for both immediate (*F*(1,28) = 3.95, *p* =.05) and long-term (*F*(1,28) = 4.29, *p* =.04) training gain indices.

**Table 3:** Means (M) and ± standard deviations (SD) for the immediate and maintenance training gains indices by group, ANOVAs results for the differences between the two groups (Trained vs. Active control group) in training gains indices. Statistically significant results are represented in bold, tendencies in bold italics

	**Immediate** **training gains**		**Maintenance training gains**		**Statistics**	
	**TG**	**AGC**	**TG**	**ACG**	**ANOVA**	*F(df) and p values*
	*M (SD)*	*M (SD)*	*M (SD)*	*M (SD)*		
CWMS accuracy	2.87 ± 2.44	0.93 ± 2.86	2.27 ± 2.31	0.47 ± 2.44	Group main effect	***F***(1,28) = 4.92, ***p*** =.035
					Gain main effect	*F*(1,28) = 2.00, *p* =.168
					Group by Gain interaction	*F*(1,28) = 0.03, *p* =.861
2-back RT	−18.03 ± 118.4	6.27 ± 142.6	−17.75 ± 126.1	−49.68 ± 73.24	Group main effect	*F*(1,28) = 0.01, *p* =.918
					Gain main effect	*F*(1,28) = 1.48, *p* =.233
					Group by Gain interaction	*F*(1,28) = 1.51, *p* =.229
2-back accuracy	−1.36 ± 8.82	0.66 ± 8.68	−0.45 ± 5.68	1.16 ± 9.10	Group main effect	*F*(1,28) = 1.68, *p* =.205
					Gain main effect	*F*(1,28) = 0.03, *p* =.955
					Group by Gain interaction	*F*(1,28) = 0.08, *p* =.778
2-back false alarms	−0.08 ± 3.82	0.41 ± 3.55	−1.50 ± 3.51	1.25 ± 3.30	Group main effect	*F*(1,28) = 2.01, *p* =.167
					Gain main effect	*F*(1,28) = 0.23, *p* =.637
					Group by Gain interaction	***F(1***,***28) = 3.38***,*** p =.076***

*CWMS *Categorization Working Memory Span task*, RT *reaction times*, TG *Training Group*, ACG *Active Control Group

#### Transfer effects

No statistically significant effects nor interactions emerged between the two groups for any of the transfer task (2-back) gain scores including RT, accuracy and false alarms, as can be seen in Table 3. Nonetheless, as there was a tendency in the false alarm Group by Gain interaction (*F*(1,28) = 3.38, *p* =.07, *η*^2^_p_ = 0.11), we decided to explore this interaction by means of planned comparisons. Between groups (TG vs. ACG) contrasts revealed that there were no significant differences for the immediate training gain index (*F*(1,28) = 0.13, *p* =.71), but the two groups differed for the maintenance training gain index (*F*(1,28) = 4.88, *p* =.03), with TG participants showing lower false alarm rates than ACG participants 6 months after the training completion.

#### Effect sizes

For the short-term, concerning training gains, a large effect size (*d* = 1.01) emerged for the CWMS in the TG. As for transfer effects, effect sizes for the 2-back (RT, accuracy, false alarms) in the TG were null or small (from 0.12 to 0.02; Table 4). In the ACG, effect sizes were small or null (from 0.25 to 0.03) for both the CWMS and the 2-back outcomes.

**Table 4:** Effect sizes for the immediate and maintenance training gains by group

	Immediate training gains		Maintenance training gains	
	Trained Group (TG)	Active Control Group (ACG)	Trained Group (TG)	Active Control Group (ACG)
CWMS accuracy	1.01	0.25	0.75	0.13
2-back RT	−0.10	0.03	−0.10	−0.20
2-back accuracy	−0.12	0.05	−0.04	0.11
2-back false alarms	−0.02	0.12	−0.45	0.46

*CWMS *Categorization Working Memory Span task*, RT *reaction times

Considering long-term benefits, the effect size for the CWMS remained large (*d* = 0.75) in the TG. Regarding transfer effects, effect sizes became closed to medium (*d* = − 0.45) for the 2-back errors, suggesting a decrease in errors between pre-test and follow-up, and remained small to null for the other *n*-back variables. Effect sizes did not vary in the ACG, except for the *2*-back errors that reached a negative closed to a medium effect size, indicating an increase in errors between pre-test and follow-up also for this group (Table 4)^3^.

### ERP data

#### Training gains

**Fig. 2:**
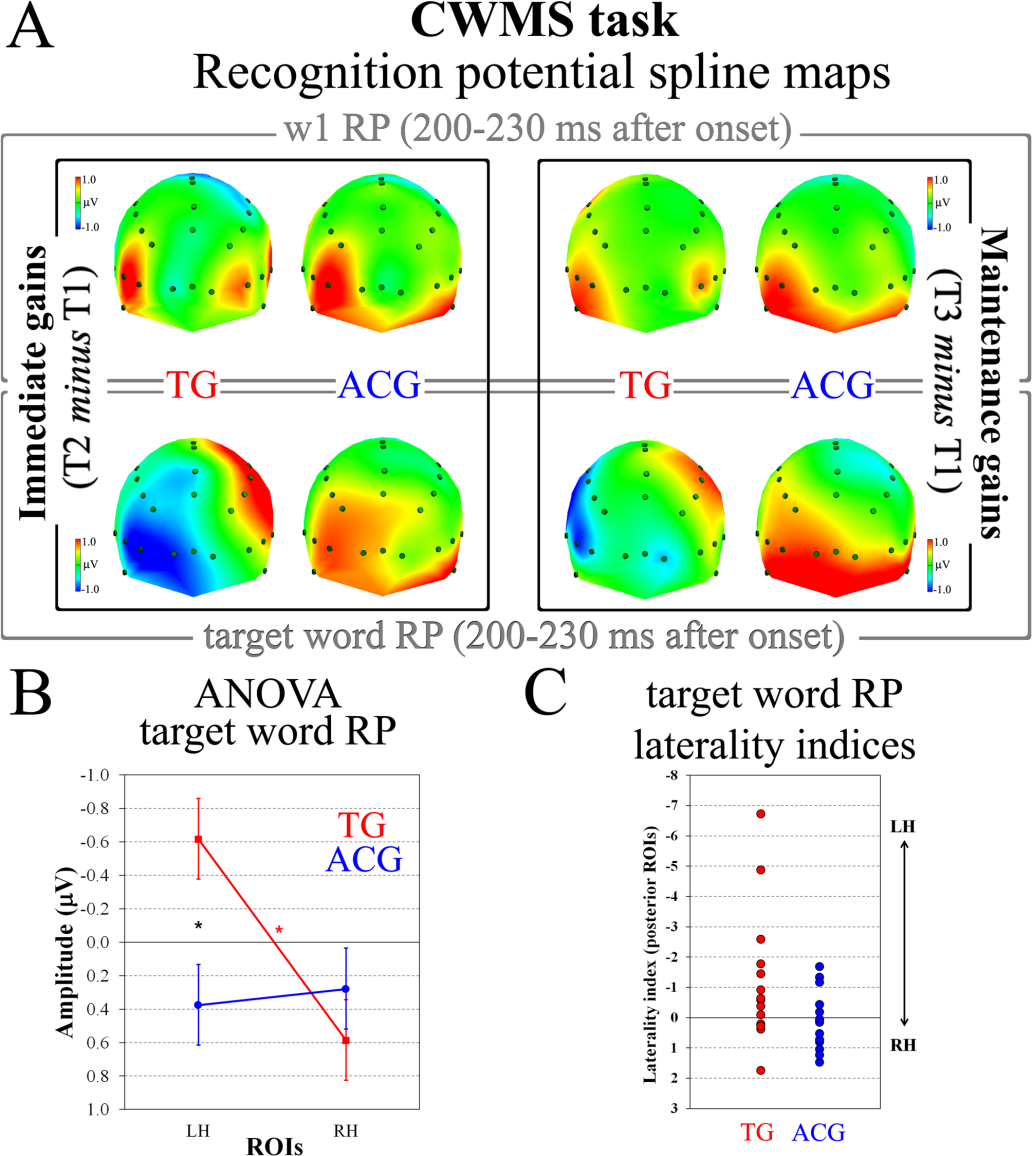
Categorization working memory span (CWMS) task: (**A**) spline-interpolated maps of the recognition potential (RP) evoked by CWMS w1 (top grey panel) and target words (bottom grey panel) as post-training (T2) minus pre-training (T1) session (i.e., the training gain effect; left black panel) and 6 months after follow-up (T3) minus pre-training (T1) session (i.e., the maintaining gain effect; right black panel) for trained group (TG) and active control group (ACG) participants. **B** ANOVA on target word RP, representing the group by laterality interaction. Vertical bars show standard errors. **C** Distribution of RP laterality indices in TG (red) and ACG (blue) participants. These indices offer a qualitative overview of leftward/rightward lateralization in the two groups: negativity values characterize greater left hemisphere (LH) activation, positive values greater right hemisphere (RH) activation * *p* <.05 * *p* <.05

Figure 2A shows the ERP spline-interpolated maps of the CWMS RP evoked by the first word (w1) and the target words that appeared at the end of each list as post-training minus pre-training (T2 – T1 session) and 6 months after follow-up minus pre-training (T3 – T1 session) for TG and ACG participants.

During the 200–230 ms time interval, spline maps revealed no RP evoked by w1 stimuli (Fig. 2A, top grey panel) for both the TG and ACG, suggesting neither training effects nor group differences. Indeed, ANOVA on w1 RP revealed no statistically significant effects (*F*(1,28) < 2.87, *p* >.101). In contrast, target words elicited the typical posterior negativity, which appears greater in the left compared with the right hemisphere after training (T2 session) in the TG only (Fig. 2A, left-bottom grey panel). This pattern was clear also 6 months after the training ended, that is, at the follow-up session (Fig. 2A, right-bottom grey panel). The pattern of target word bilateral activation the ACG showed appeared unchanged throughout time (Fig. 2A, bottom grey panel). The ANOVA conducted on the 200–230 ms time interval of immediate (T2 – T1 session) and maintenance (T3 – T1 session) training gain indices on the RP amplitude of target words revealed a significant two-way group by laterality interaction (*F*(1,28) = 4.60, *p* =.04, *η*^2^_p_ = 0.14). As seen in Fig. 2B, regardless of training gain index (immediate or maintenance), TG participants showed significantly greater left versus right RP (*p* <.05), whereas no differences were found between left and right ROIs, that is, the RP was not left lateralized, in ACG (Fig. 2C). Furthermore, considering group differences, the TG exhibited significantly greater RP amplitude than ACG participants in left (*p* <.05) but not in right ROIs.

To test the reliability of results in both immediate and maintained training gain indices, planned comparisons have been carried out on TG (left vs. right laterality effect) and between groups (TG vs. ACG in left ROIs). Results revealed that the TG laterality effect was significant for both immediate (*F*(1,28) = 6.78, *p* =.014) and maintenance (*F*(1,28) = 4.55, *p* =.041) training gain indices, whereas greater RP amplitude in left ROIs of TG versus ACG participants was significant for immediate (*F*(1,28) = 5.82, *p* =.022) training gain index, but not for the maintenance one (*F*(1,28) = 2.02, *p* =.17).

#### Transfer effects

**Fig. 3:**
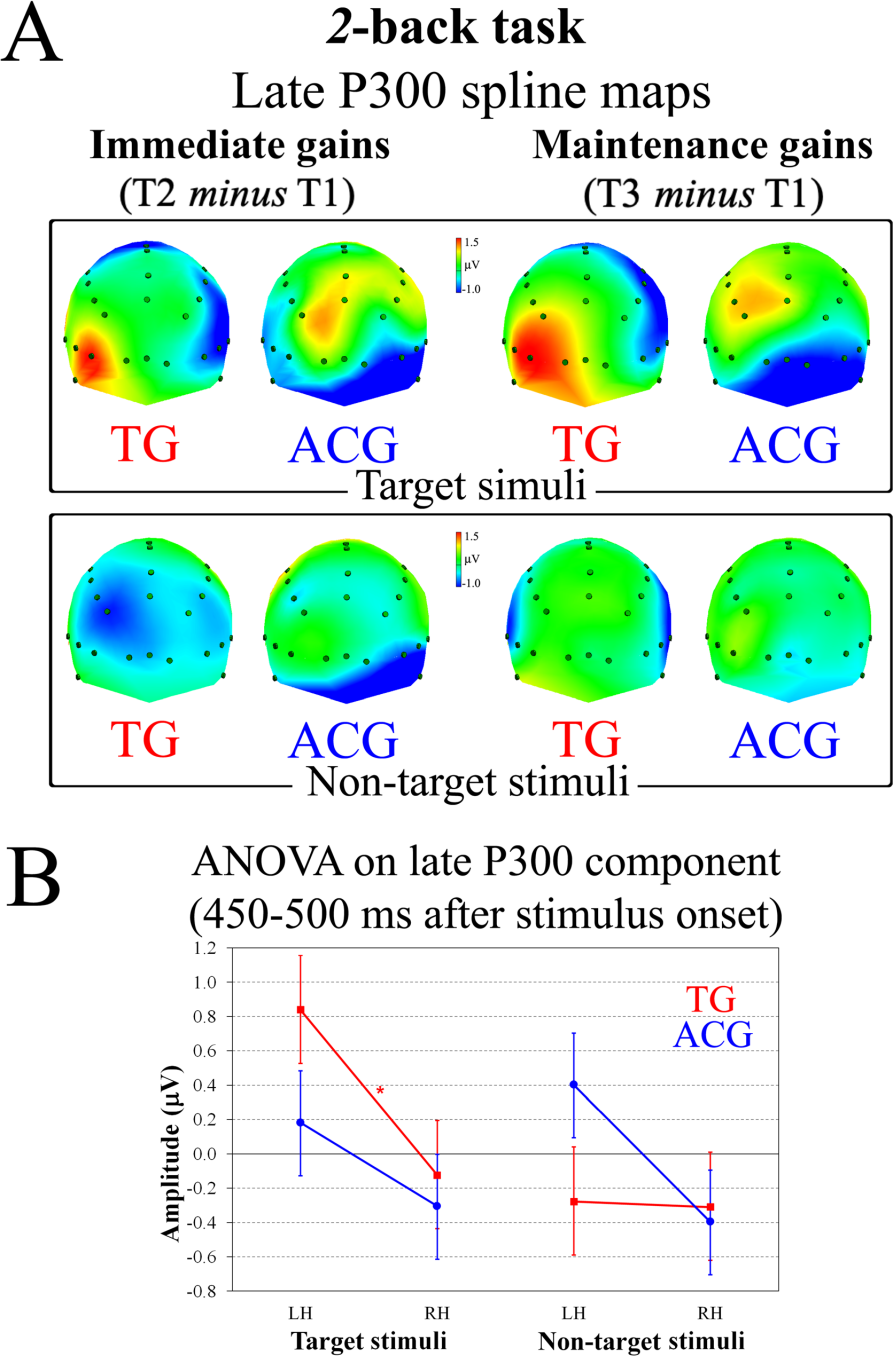
*2*-back task: (**A**) Spline-interpolated maps of the late P300 evoked by Target stimuli (top panel) and Non-target stimuli (bottom panel) as post-training (T2) minus pre-training (T1) session (i.e., the immediate training gain effect; left panels) and 6 months after follow-up (T3) minus pre-training (T1) session (i.e., the maintenance training gain effect; right panels) for training group (TG) and active control group (ACG) participants. **B** ANOVA on late P300, representing the group by stimulus by laterality interaction. Vertical bars show standard errors * *p* <.01

*2-back task.* Figure 3A shows the ERP spline-interpolated maps of a late P300 component evoked by *2-back* task Target and Non-target stimuli as post- minus pre-training (T2 – T1 session) and follow-up minus pre-training (T3 – T1 session) for TG and ACG participants.

The ERP spline-interpolated maps revealed positive activations in left versus right hemisphere for Target stimuli during the late P300 (450–500 ms) temporal interval after training (T2 session) in TG but not ACG participants (Fig. 3A, left top panel). This pattern was also clear 6 months after the training ended (Fig. 3A, right top panel). In contrast, Non-target stimuli elicited bilateral activity regardless of participants’ group (Fig. 3A, bottom panel). The ANOVA conducted on the 450–500 ms time interval of immediate (T2 – T1 session) and the maintenance (T3 – T1 session) training gain indices on the late P300 amplitude revealed a significant three-way Group by Stimulus by Laterality interaction (*F*(1,28) = 5.51, *p* =.02, *η*^2^_p_ = 0.17). As seen in Fig. 3B, regardless of gain (immediate or maintained), TG participants showed significantly greater left versus right P300 (*p* <.01) after Target stimulus onset, whereas no differences were found between left and right ROIs in the ACG. No significant laterality or group differences were found for Non-target stimuli.

To test the reliability of this results pattern in both immediate and maintenance training gain indices, planned comparisons were conducted on the TG (left vs. right laterality effect) for Target stimuli only. Results revealed that the TG laterality effect was significant for both immediate (*F*(1,28) = 3.27, *p* =.05) and maintenance (*F*(1,28) = 8.06, *p* =.008) training gain indices.

**Fig. 4:**
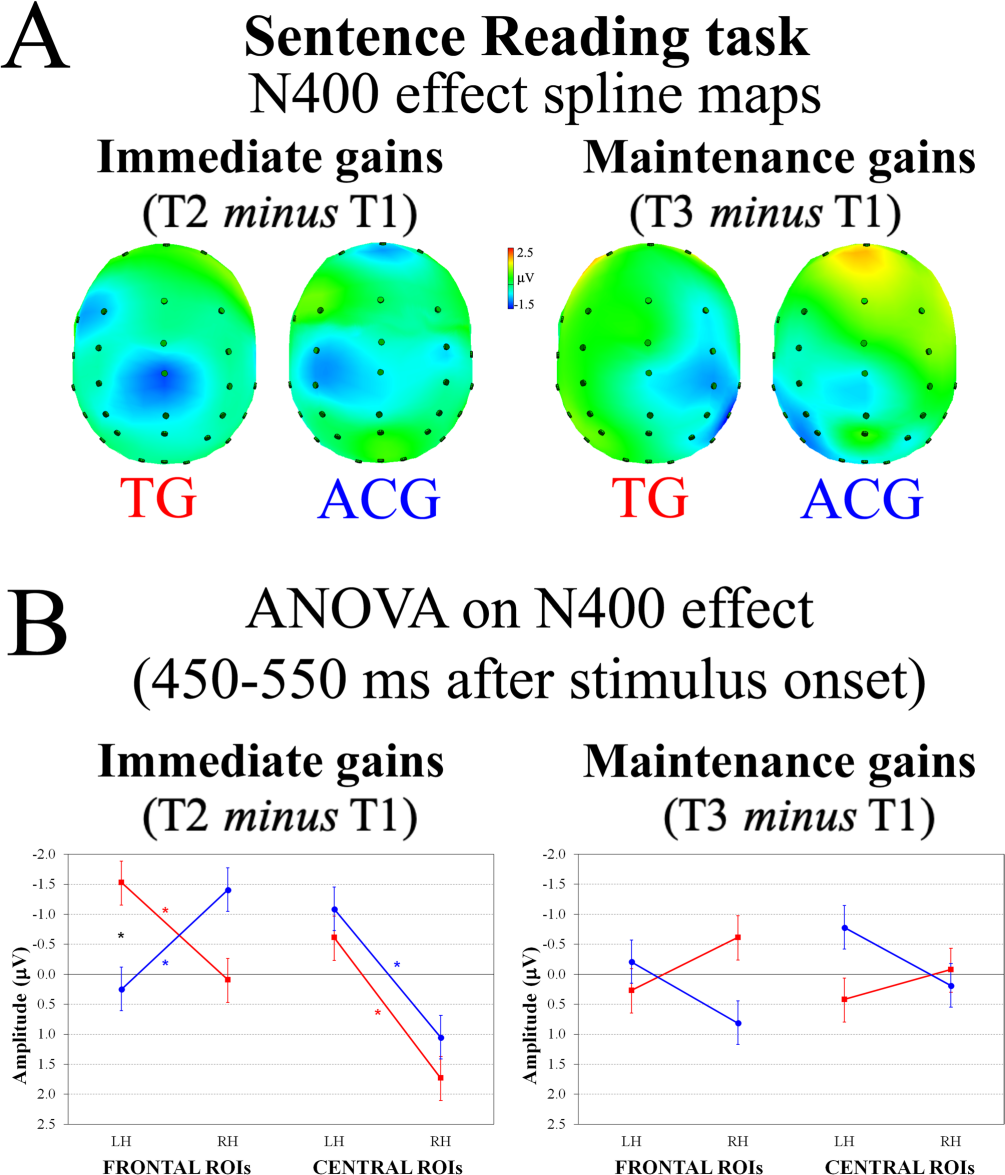
Sentence-reading task: N400 effect as mismatching minus matching stimuli. **A** Spline-interpolated maps of the N400 effect as post-training (T2) minus pre-training (T1) session (i.e., the immediate training gain effect; left panel) and 6 months after follow-up (T3) minus pre-training (T1) session (i.e., the maintenance training gain effect; right panel) for training group (TG) and active control group (ACG) participants. **B** ANOVA on N400 effect, representing the group by gain by region by laterality interaction. Vertical bars show standard errors * *p* <.05 * *p* <.05 * *p* <.05

*Sentence-Reading task.* Figure 4A shows the ERP spline-interpolated maps of the N400 component evoked by mismatch minus match stimuli as post- minus pre-training (T2 – T1 session) and 6 months after follow-up minus pre-training (T3 – T1 session) for TG and ACG participants.

The ERP spline-interpolated maps showed the N400 component in left frontal sites as well as at the vertex (Cz) during the 450–550 ms time interval after training (T2 session) in TG participants, whereas ACG adults exhibited greater N400 in left centro-parietal sites (Fig. 4A, left top panel). This pattern dramatically changed 6 months after the training ended, as TG participants revealed greater N400 in right centro-parietal sites, and ACG in left posterior sites (Fig. 4A, right top panel). The ANOVA conducted on the 450–550 ms time interval of immediate (T2 – T1 session) and maintenance (T3 – T1 session) training gain indices on the N400 component (mismatch minus match stimuli) revealed a significant four-way Group by Gain by Region by Laterality interaction (*F*(1,28) = 4.67, *p* =.03, *η*^2^_p_ = 0.14). As can be seen in the left panel of Fig. 4B, considering immediate training gain (T2 minus T1) index effects, TG participants showed significantly greater left versus right N400 effect at both frontal and central ROIs (*p* <.05 and *p* <.01, respectively), whereas ACG participants revealed significantly greater right versus left N400 effect at frontal ROIs (*p* <.05) and left versus right N400 effect at central ROIs (*p* <.01). Groups showed different N400 effects only in frontal left ROI (*p* <.05). Considering the maintained training gain index (T3 minus T1; Fig. 4B, left panel), post hoc tests provided no significant laterality or group differences.

## Discussion

The present pilot study aimed to assess the efficacy of a brief WM training in older adults considering not only participants’ behavioral performance, but also the electrophysiological changes driven by such a brief training procedure. For the first time, here we attempted to more comprehensively ascertain both short- and long-term WM training gains and transfer effects, and the cortical changes associated with behavioral ones. Applying a double-blind experimental protocol, behavior and cortical mechanisms underlying the benefits of WM training were in fact examined not only in a WM task similar to the ones directly trained (training gains), but also in untrained ones (i.e., transfer effects). Training benefits were, then, assessed immediately after the training completion (immediate training gains), as typically done, but also 6 months after the training conclusion (maintenance training gains).

At the behavioral level, our results showed that the two groups differed in their immediate and maintenance training gains indices in the criterion task (CWMS) in favor of the TG. These results (supported by the large effect sizes found for the TG but not for the ACG) indicated that both at short- and long-term participants in the TG significantly improved their performance, compared with the ACG, and maintained such a gain at the 6-months follow-up. Overall, such a pattern of findings aligns with previous evidence highlighting how WM training could foster short- and, though rarely examined, also long-term training gains in WM tasks similar to those trained [7, 10, 11, 23].

In line with the behavioral training gains, the analysis carried out on electrophysiological data revealed that target words elicited greater word RP in posterior left ROIs between pre-test and post-test in the TG only. In line with our past study [25], the WM involvement appears at early phases of word processing as a left-lateralized pattern of activation specific for target words, given that no group or laterality effects were found for the first word of each list, used as a control condition. Notably, this left-lateralized word RP also persisted between the pre-test and the follow-up assessment, carried out 6 months after the WM training completion. Such a significant change in the posterior left ROI activation support the plasticity of cortical mechanism induced by the training. It might also represent the underlying behavioral mechanism related to long-term CWMS -specific- benefits following the WM training. However, this is just a speculation that merits to be verified by other studies using the same procedure and running correlational analyses with a robust sample size.

However, contrary to our expectations and previous literature [7, 10], the WM training did not provide clear behavioral transfer effects to the *2*-back task. The two groups did not differ in their immediate training gain indices for the *2*-back RT, accuracy and false alarms, as the -small- effect sizes also confirmed.

These results could be due to the demands and processes characterizing the *2*-back, which mainly involves updating processes, differently from the trained tasks also in terms of the degree of attentional control or the involvement of retrieval processes from long-term memory [30, 52]. Such differences could have prevented trained participants from manifesting the behavioral benefits from the intervention in terms of generalized benefits to such an untrained task, at the short term. Nonetheless, results from planned comparisons for the false alarms in the *2*-back task revealed that the two groups differed in their maintained gain scores, with -medium- effect sizes suggesting a decrease in the number of false alarms for the TG (and an increase of false alarms for the ACG). This result should be obviously interpreted with caution, given the limited sample size and the tendency to statistical significance of the Group by Gain interaction (*p* =.07), but it might represent the so-called “sleeper effect” phenomenon (when benefits more likely emerge in the long term), which is not new in WM training literature in aging, but should still be clarified [53]. In general, this effect suggests that, in some abilities, the benefits of WM training might take longer to be detected, at the behavioral level. They thus implicitly underscore the importance of systematically including follow-up and brain-related assessments to capture better the nature of benefits that such cognitive interventions provide in older adults. Indeed, unlike the behavioral findings, the analysis carried out on electrophysiological data revealed that Target stimuli elicited greater P300 amplitude in posterior left ROIs in the TG only. Notably, this transfer effect appeared not only as soon after the WM training ended (between the pre-test and post-test), but also 6 months after the training completion (between the pre-test and the follow-up). Notwithstanding the lack of behavioral effects, in our sample, the WM training effectively induced significant cortical changes in the attentional resources addressed to the target (but not to the irrelevant) stimulus processing. In other words, the increased P300 amplitude unveiled the training benefit in target processing, in TG participants only, immediately after the training and at the long term. Probably, there is a sort of mismatch between cortical and behavioral changes, which need more time to be detected and manifested. The abovementioned tendency towards a decrease in the number of false alarms found for TG at the follow-up apparently supported this interpretation. However, the lack of a second follow-up (e.g., 1 year after the WM training ended) prevents verifying changes in the *n*-back accuracy, thus validating such a speculation. Future studies should make the effort of combining behavioral and brain-related analyses, including repeated follow-up sessions to clarify this critical issue.

Nevertheless, the dissociation between our behavioral and electrophysiological results pinpoints the importance of considering not only participants’ performance, but also their cortical functioning to ascertain the efficacy of WM training among older adults and its related changes. This should be done not only because they could provide complementary information, but also because they could contribute to unveil the plasticity pattern that might not be immediately captured considering behavioral performance only [54].

In addition, similar to the CWMS RP left-lateralized post-training activation, the *2*-back task P300 component reached the maximum amplitude in the posterior left ROI – a pattern that was not found in past studies, where the *n*-back was used to train participants during the training sessions [19, 55]. As in our WM training sets of presented audio-recorded words and participants recalled target words (also tapping with their hand on the table when they heard the name of an animal), it is not surprising that after such a WM training regimen, greater left hemisphere activation appeared in both TG participants’ target word processing (in the CWMS task) and their Target stimulus/letter processing (represented by consonants in our *2*-back task). Notably, this lateralized pattern persisted 6 months after the training conclusion, thus revealing that the effect of verbal WM practice induced a long-term plastic change in the cortical activation within language-related circuits. Considering past literature on physiological brain activation in aging, and Cabeza’s [55, 56] Hemispheric Asymmetry Reduction in OLDer adults (HAROLD) model in particular, less lateralized or even bilateral brain distribution was found in older compared to younger adults in various tasks, including WM ones [15, 57]. These age-related decreased hemispheric differences have been attributed to the recruitment of homologous areas in the nondominant hemisphere, and they have been interpreted as an effort to compensate for losses in the dominant hemisphere [58] or aging-induced dedifferentiation [58, 59] in a physiological (i.e., typical) aging process. As above-mentioned, the leftward asymmetry found in the present study may directly depend on the characteristics of the WM task used for training, considering the CWMS task represented a challenging and complex condition involving an in-depth word analysis. Because language shows left hemisphere dominance for about 95% of right-handed individuals and 70–85% of left-handers [48, 60], the training probably stimulates the linguistic network and induced a long-lasting laterality effect.

This pattern of post-training leftward asymmetry also appeared in the other task administered to ascertain transfer effects, broadening to the semantic processes involved in reading comprehension (Sentence Reading task). A semantically inappropriate word in a sentence-reading task elicited an N400 component showing significant left hemisphere lateralization both at frontal and central ROIs in TG participants between the pre-test and post-test, whereas it revealed a right lateralization in frontal ROIs and left lateralization in central ROIs in ACG participants. While the engagement of central sites of left hemisphere is typical – especially considering fMRI results [61] – for this task, the between-group inverted lateralization suggested a training effect in TG participants’ frontal regions. According to Van Petten and Luka [61], the activation of left inferior frontal gyrus (IFG) depends on strategic, task-related processes, including passive-reading paradigms. Typically, the left IFG is engaged in the controlled retrieval of lexical contents, regardless of the specific experimental condition (i.e., whether the final word matches the prior context [61]). Again, regarding the CWMS and *2*-back tasks, after WM training, greater left hemisphere activation appeared in TG participants’ target word processing (represented by the mismatching final word in our sentence-reading task), at both frontal and central ROIs. However, unlike the other two tasks, this effect appeared after the training but was not maintained, because 6 months after the training ended no group or laterality effects were found. The lack of long-lasting effects may depend on a key feature of the sentence-reading task. Indeed, this is a passive reading task in which the brain automatically completes each sentence with only one semantically appropriate final word. This automatic process is probably restored after the benefits gained soon after the WM training. This is not a typical WM task, but it represents something rather different from the exercises conducted during the training sessions (word processing and memory maintenance, followed by word rehearsal and recall).

Despite these interesting results, the present pilot study has some limitations.

The quite small sample size prevents us to obtain a comprehensive picture of the behavioral and cortical effects, and their correlates, provided by the WM training proposed, but the collection of both behavioral and electrophysiological data in a long temporal window, including a 6-month follow up session should at least partially justify and mitigate this issue.

Though among the strength of our pilot study there is the fact that we used a double-blind repeated-measures experimental design, having also different experimenters in charge of administering either the training to the TG or the alternative activities to the ACG would have allowed to ensure a clear blinding, an aspect that merits to be accounted in future studies. Future studies, beyond an active control group, should also include a passive control condition to further confirm the behavioral and physiological effects of WM training among older adults, also controlling for any potential “expectations” effect.

We did not use a hearing-screening task, which could be recommended for auditory cognitive training. However, some precautions were adopted, such as the use of a sound amplifier, and the participants were asked if they could easily hear the training stimuli during the experimental sessions.

Despite the effort of ascertaining the presence transfer effects with paradigms that are also suitable for collecting physiological data, the sentence reading task used here was not comprehensively informative of both the behavioral and physiological changes provided by the WM training. In addition, the choice to administer two different transfer tasks (i.e., the *2*-back and the sentence reading task) together with the trained tasks (i.e., the CWMS task) resulted in long electrophysiological experimental sessions. To control for this critical issue, we decided to administer the minimum number of stimuli for each task and conditions, to obtain reliable electrophysiological data. As a consequence, we decided to average all good trails, regardless of their later behavioral performance (if any). Future studies should therefore make the effort of including a more fine-grained battery of tasks capable of better capturing the generalizability of any benefits provided by WM training in aging both at the behavioral and physiological levels, also at long-term, as done here with a 6-month follow-up session (rarely used in training studies, even those focusing on the behavioral data only). In turn, this will allow researchers to increase the number of trials included in each task and condition, and to exclude from ERP analysis all the trials associated with behavioral errors.

Finally, a better understanding of the role of individual differences, for instance in terms of socio-demographic characteristics (e.g., age, gender, education), in influencing WM training benefits is warranted, especially in studies that involve also electrophysiological data.

Notably, the examination of WM training changes not only at behavioral but also at cortical level, at both short- and long term, confirms that aging is characterized by a certain degree of plasticity, which can emerge at different timepoints (either post-test or at the follow-up) and levels (behavioral and/or cortical), depending on the processes involved. Engaging older adults in WM training activities can effectively promote behavioral changes and foster the range of neural activity of the WM circuitry [62], particularly in the left hemisphere.

## Supplementary Information

Supplementary Material 1.

## Data Availability

The data that support the findings of this study are available on request from the corresponding authors. The data are not publicly available, due to the confidential nature of (part of) the dataset we collected.

## Footnotes

1 An a-priori power analyses (G*Power) showed that a total sample size of 28 participants sufficed to obtain a power of 0.80 in a repeated measures ANOVA, within and between interaction, with a medium effect size (F = 0.25) and a significance level of α = 0.05. Considering the analyses conducted for behavioral data, a sample size of 21 participants per group is needed to obtain a power of 0.80 in a *t*-test difference between two independent means with a large effect size (*d* = 0.80) and a significance level of α = 0.05. It is to note that we initially recruited around 50 eligible participants who agreed to participate in the study. Because of the training procedure, participants were gradually involved in the study (4–5 participants every two weeks). A covariate adaptive randomization was used, gradually scrolling the initial list, to ensure, also due to drop-outs (i.e., people who withdrew before starting the protocol since they were no longer available or withdrew during the pre-test assessment due to personal issues or worries and discomfort with the EEG measurements), that the two groups were balanced in terms of participants’ characteristics (age, gender and years of formal education). Overall, 39 participants (TG = 20, CG = 19) completed the pre- and post-test assessments, however 9 participants (TG = 5, CG = 4) were no longer available to complete the follow-up assessment due to personal issues. Therefore, 30 participants (TG = 15, CG = 15) completed the whole study protocol. Participants’ allocation was never shared with the two experimenters who conducted the assessment sessions until the very end of the research protocol (at the end of the follow-up session) when participants were told about the aims of the study.

2 Practice trials of CWMS task were included in analyses to enlarge the number of trials to be analyzed using ERPs. They were identical to the experimental trials, but participants were told that some practice trials, to familiarize them with the task on computer (and for the experimenter to be sure that they correctly understood how to complete the task on computer according to the instructions and the familiarization received), were presented. The experimenter was instructed to repeat the computerized practice trials if any participant’s uncertainty in how to complete the task according to the instructions received emerged. None of the participants had issues in completing the practice trials, therefore the practice trials were never repeated.

3 To ascertain any significant changes between the time points considered (pre-test vs. post-test and pre-test vs. follow-up), *t*-test separately for each group was also run. Results, in line with the Cohen’s *d*, showed that only for TG there was a significant improvement in the CWMS performance, both at short-term -from pre-test to post-test- (*t* = 4.54, *p* <.001) and at long-term -from pre-test to follow-up- (*t* = 3.795, *p* <.001).
