# Supplementary material for: Short- and long-term cognitive and electrophysiological effects of a brief working memory training in older adults: a pilot study
Source: BMC Geriatr. 2025 Nov 7;25:856. doi: 10.1186/s12877-025-06507-2 (PMC12595693; doi:10.1186/s12877-025-06507-2)
Supplement: Supplementary file 1 — Supplementary Material 1. [file 12877_2025_6507_MOESM1_ESM.docx]

*Supplementary materials for*

**Short- and long-term cognitive and electrophysiological effects of a brief working memory training in older adults: A pilot study**

Erika Borella, Elena Carbone, Chiara Spironelli

**Part I – Details of rejected trials disaggregated by group and conditions**

In the next Tables, the percentages of rejected trials for each group, stimulus and experimental session separately for each task, and the statistical analyses performed to evaluate the presence of any significant differences are presented.

**Table S1.** Mean percentage of trials rejected in the Categorization Working Memory Span (CWMS) task.

| **Task – condition** | **Group** | **Session** | | | **Mean %** |
| --- | --- | --- | --- | --- | --- |
|  |  | **Pre-training** | **Post-training** | **Follow-up** |  |
| CWMS – w1 stimuli | ACG | 27.56% | 25.61% | 23.82% | 26.49% |
|  | TG | 25.53% | 25.53% | 30.91% |  |
| CWMS – target stimuli | ACG | 33.09% | 25.80% | 24.08% | 26.50% |
|  | TG | 25.51% | 25.79% | 24.75% |  |
| **Statistical analysis** |  |  |  | ***F*** | ***p* value** |
| Group main effect |  |  |  | 0.003 | 0.953 |
| Stimulus main effect |  |  |  | 0.001 | 0.992 |
| Session main effect |  |  |  | 1.600 | 0.211 |
| Group by Stimulus interaction |  |  |  | 1.323 | 0.256 |
| Group by Session interaction |  |  |  | 3.181 | 0.096 |
| Group by Stimulus by Session interaction | |  |  | 0.382 | 0.684 |

*ACG = Active Control Group*

*TG = Trained Group*

**Table S2.** Mean percentage of trials rejected in the *2*-back task.

| **Task – condition** | **Group** | **Session** | | | **Mean %** |
| --- | --- | --- | --- | --- | --- |
|  |  | **Pre-training** | **Post-training** | **Follow-up** |  |
| *2*-back – Go stimuli | ACG | 23.83% | 17.67% | 23.13% | 24.31% |
|  | TG | 25.31% | 26.46% | 28.45% |  |
| *2*-back – No-Go stimuli | ACG | 24.46% | 19.52% | 23.44% |  |
|  | TG | 26.01% | 24.76% | 28.66% |  |
| **Statistical analysis** |  |  |  | ***F*** | ***p* value** |
| Group main effect |  |  |  | 1.137 | 0.295 |
| Stimulus main effect |  |  |  | 0.072 | 0.790 |
| Session main effect |  |  |  | 0.799 | 0.455 |
| Group by Stimulus interaction |  |  |  | 0.232 | 0.634 |
| Group by Session interaction |  |  |  | 0.404 | 0.670 |
| Group by Stimulus by Session interaction | |  |  | 0.500 | 0.611 |

*ACG = Active Control Group*

*TG = Trained Group*

**Table S3.** Mean percentage of trials rejected in the Sentence Reading task.

| **Task – condition** | **Group** | **Session** | | | **Mean %** |
| --- | --- | --- | --- | --- | --- |
|  |  | **Pre-training** | **Post-training** | **Follow-up** |  |
| SR – Congruent stimuli | ACG | 24.85% | 23.36% | 23.22% | 22.83% |
|  | TG | 16.14% | 23.34% | 24.47% |  |
| SR – Incongruent stimuli | ACG | 24.65% | 20.38% | 24.98% |  |
|  | TG | 18.50% | 22.16% | 27.91% |  |
| **Statistical analysis** |  |  |  | ***F*** | ***p* value** |
| Group main effect |  |  |  | 0.124 | 0.727 |
| Stimulus main effect |  |  |  | 0.346 | 0.561 |
| Session main effect |  |  |  | 1.032 | 0.363 |
| Group by Stimulus interaction |  |  |  | 1.220 | 0.279 |
| Group by Session interaction |  |  |  | 1.565 | 0.220 |
| Group by Stimulus by Session interaction | |  |  | 0.025 | 0.976 |

*SR = Sentence Reading task*

*ACG = Active Control Group*

*TG = Trained Group*

**Part II – Waveforms for groups, tasks and conditions**

In the next Figures, we showed the grand-mean waveforms of electrodes selected for statistical analyses separate for sessions (i.e., pre-training or T1, post-training or T2, and follow-up or T3; panel A of each figure) and the difference waves to highlight the training gain indices (i.e., immediate gain or T2–T1 difference, and maintained gain or T3–T1 difference; panel B of each figure) for each group, stimulus, and task.


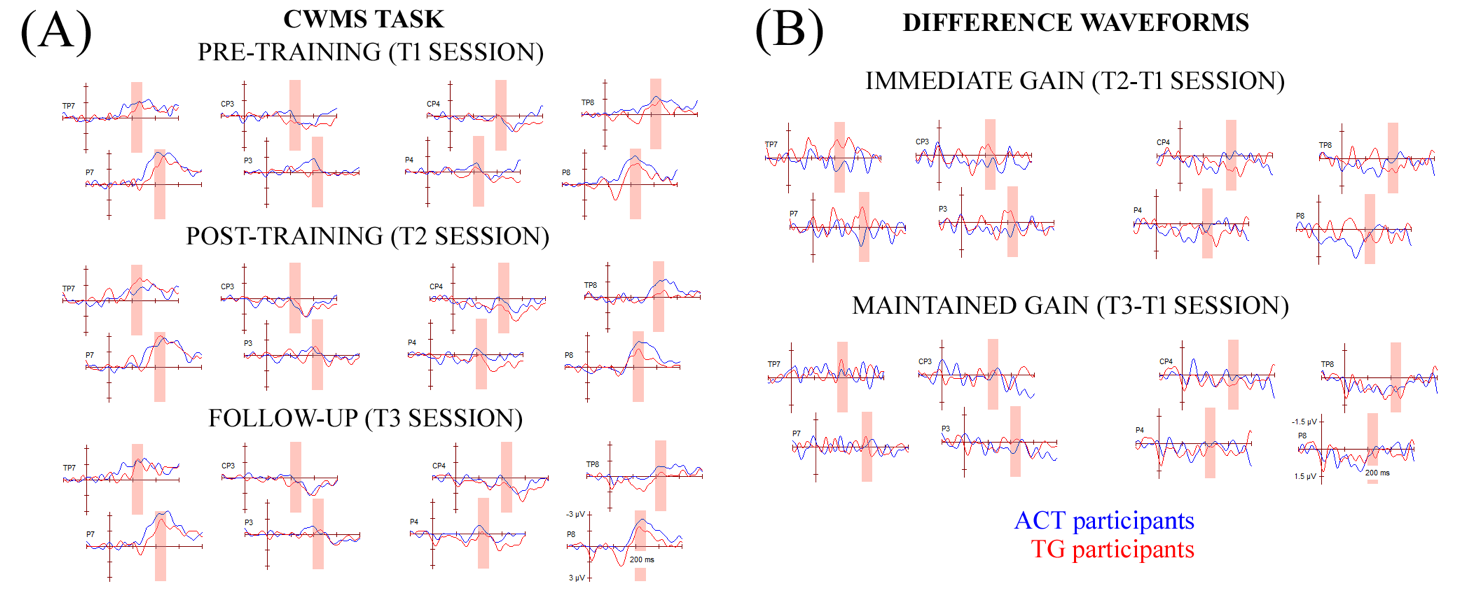


**Figure S1.** CWMS task: the pink color corresponds to the word RP peak, about 200–230 ms after TARGET stimulus onset. (A) Grand-average waveforms of the 4 electrodes included in the posterior left (i.e., CP3, P3, P7, TP7) and right (i.e., CP4, P4, P8, TP8) regions of interest (ROIs) for ACT (blue color) and TG (red color) participants during the pre-training (T1 session, top row) post-training (T2 session, central row) and follow-up (T3 session, bottom row) recording. (B) Difference waves showing the gain effects in each electrode of the ROIs, and group.

**
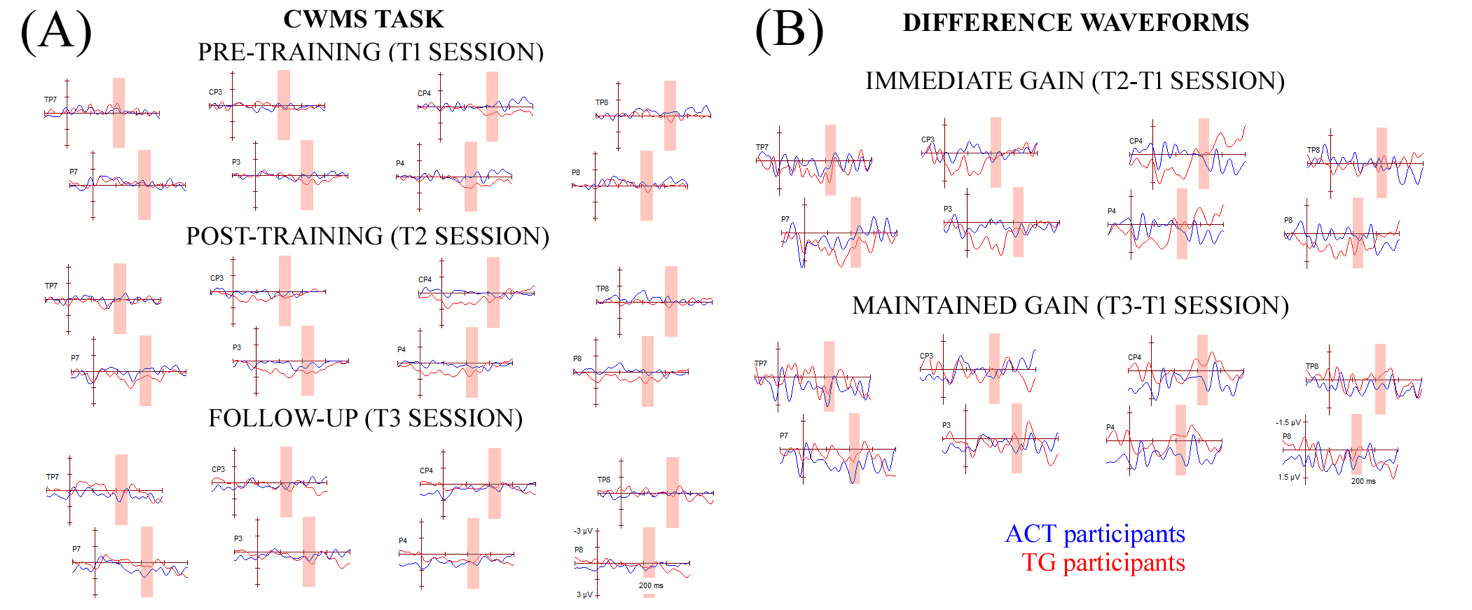
**

**Figure S2.** CWMS task: the pink color corresponds to the word RP peak, about 200–230 ms after W1 stimulus onset (CONTROL CONDITION). (A) Grand-average waveforms of the 4 electrodes included in the posterior left (i.e., CP3, P3, P7, TP7) and right (i.e., CP4, P4, P8, TP8) regions of interest (ROIs) for ACT (blue color) and TG (red color) participants during the pre-training (T1 session, top row) post-training (T2 session, central row) and follow-up (T3 session, bottom row) recording. (B) Difference waves showing the gain effects in each electrode of the ROIs, and group.

**
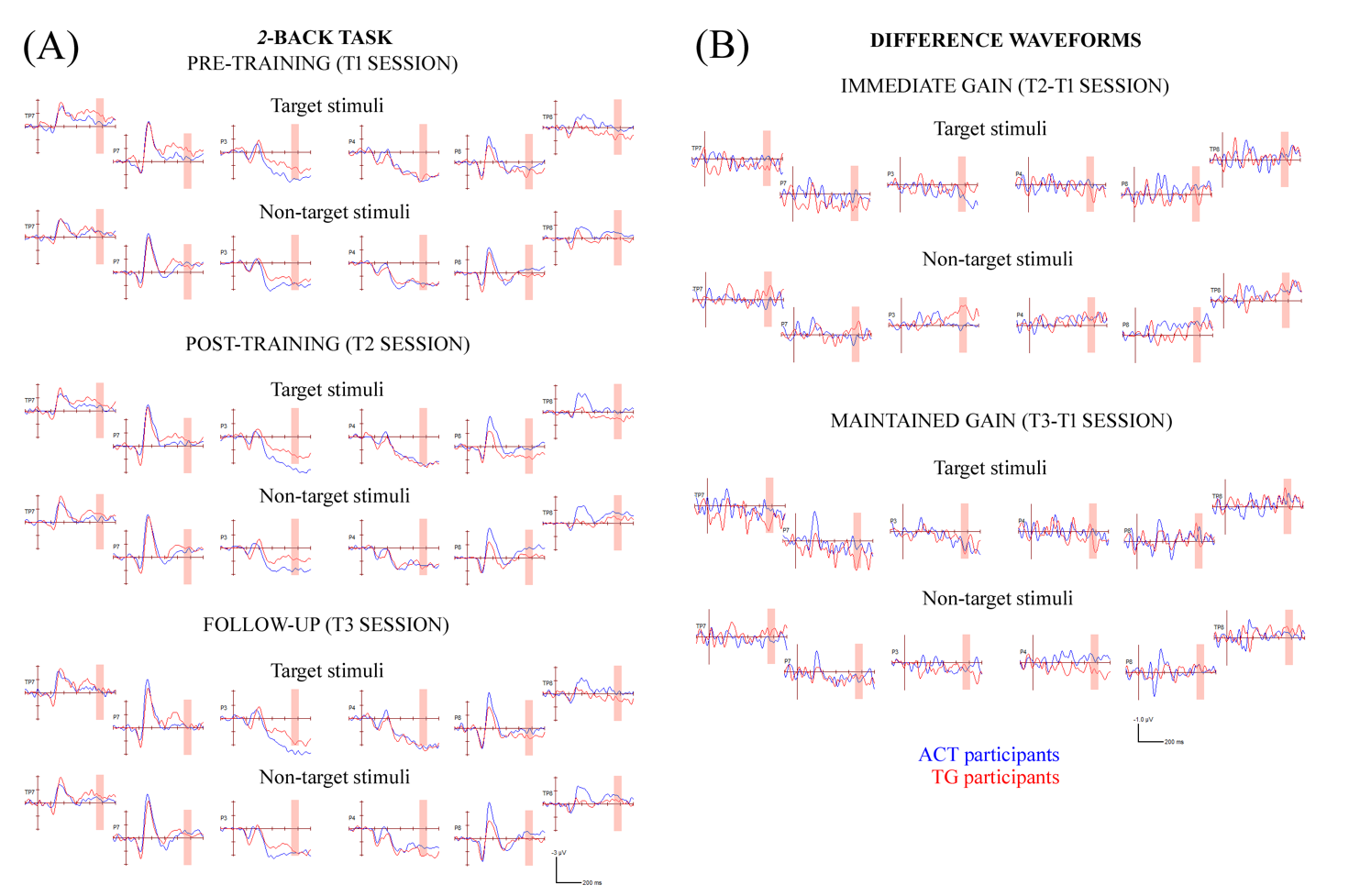
**

**Figure S3.** *2*-back task: the pink color corresponds to the P300 component, about 450–500 ms after stimulus onset. (A) Grand-average waveforms of Target and Non-target stimuli, including the 3 electrodes of the posterior left (i.e., P3, P7, TP7) and right (i.e., P4, P8, TP8) regions of interest (ROIs) for ACT (blue color) and TG (red color) participants during the pre-training (T1 session, top row) post-training (T2 session, central row) and follow-up (T3 session, bottom row) recording. (B) Difference waves showing the gain effects in each electrode of the ROIs, group and stimulus condition.

**
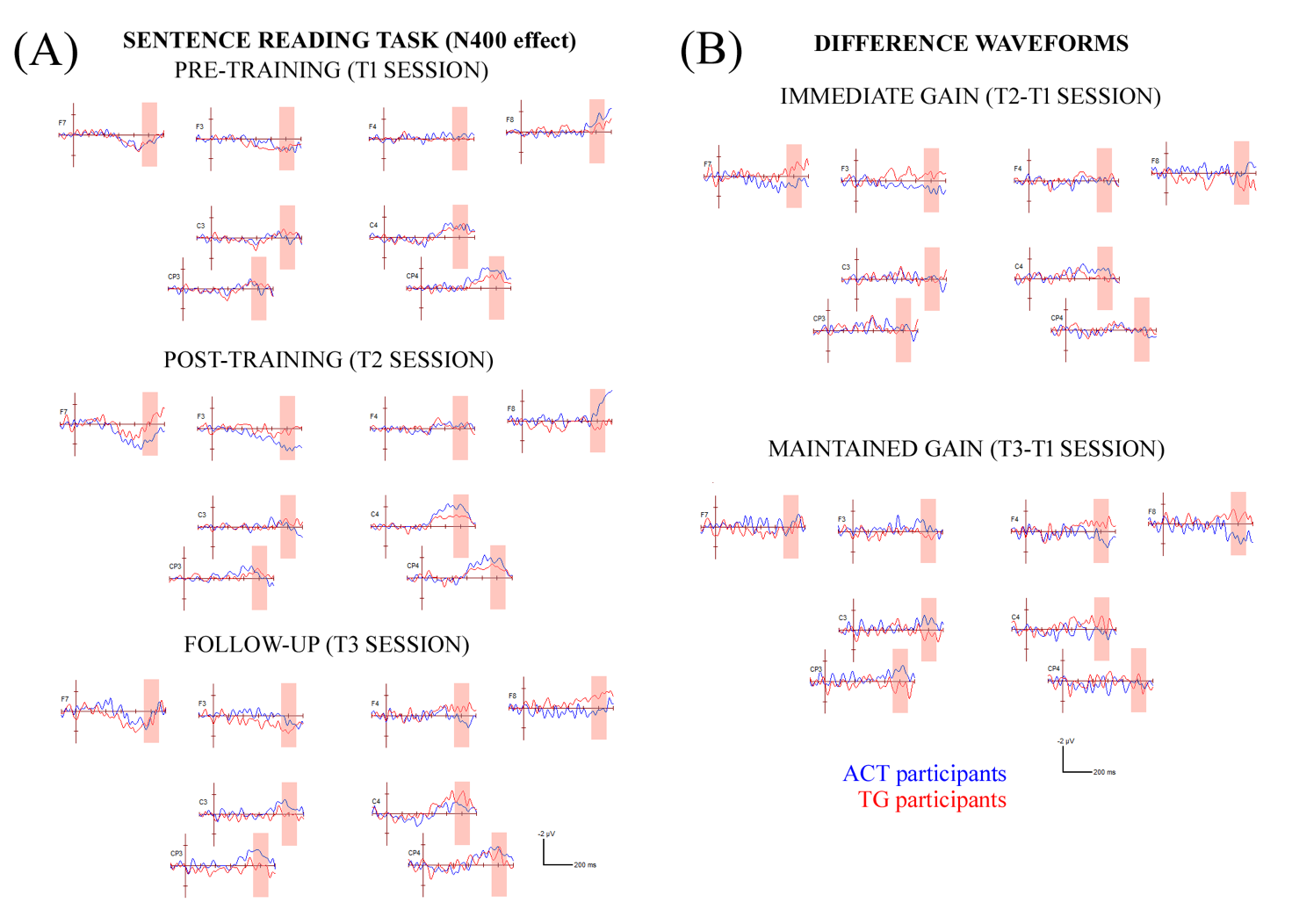
**

**Figure S4.** Sentence reading task: the pink color corresponds to the N400 effect (computed as the Incongruent minus Congruent stimulus difference), about 450–550 ms after stimulus onset. (A) Grand-average waveforms of the 2 electrodes included in the frontal left (i.e., F3, F7) and right (i.e., F4, F8) regions of interest (ROIs), as well as in the central left (i.e., C3, CP3) and right (i.e., C4, CP4) ROIs for ACT (blue color) and TG (red color) participants during the pre-training (T1 session, top row) post-training (T2 session, central row) and follow-up (T3 session, bottom row) recording. (B) Difference waves showing the gain effects in each electrode of the ROIs, and group.

**Part III – Descriptive statistics by group and assessment occasions, and preliminary analyses of pre-training (i.e., T1 session) baseline condition between the two groups**

Descriptive statistics [Means (*M*) and Standard Deviations (*SD*)] for behavioral outcomes at the pre-test, post-test and follow-up assessment sessions by group are reported in Table S4. We performed a between-group *t* test on the pre-training (T1 session), to test for possible *a priori* differences (Table S4). As can be seen in Table S4, no between-group differences were found in the pre-training (T1 session).

**Table S4.** Descriptive statistics [Means (*M*) and Standard Deviations (*SD*)] for the measures of interest at the pre-, post-test and follow-up by group, and t-test results for the differences between the two groups (Trained Group *vs.* Active Control one) at pre-test.

|  | **Pre-test** | | | | **Trained Group *vs.* Active Control Group** | | **Post-test** | | | | **Follow-up** | | | |
| --- | --- | --- | --- | --- | --- | --- | --- | --- | --- | --- | --- | --- | --- | --- |
|  | **Trained Group** | | **Active Control Group** | |  |  | **Trained Group** | | **Active Control Group** | | **Trained Group** | | **Active Control Group** | |
|  | ***M*** | ***SD*** | ***M*** | ***SD*** | ***t_(1,28)_*** | ***p*** | ***M*** | ***SD*** | ***M*** | ***SD*** | ***M*** | ***SD*** | ***M*** | ***SD*** |
| CWMS accuracy | 12.07 | 3.24 | 12.60 | 3.97 | -0.40 | 0.69 | 14.93 | 2.15 | 13.53 | 3.11 | 14.33 | 2.52 | 13.07 | 3.03 |
| 2-back RT | 781.03 | 168.05 | 756.16 | 237.14 | 0.33 | 0.74 | 762.99 | 182.01 | 762.44 | 197.74 | 763.28 | 167.49 | 706.48 | 234.25 |
| 2-back accuracy | 76.59 | 10.62 | 74.66 | 10.51 | 0.45 | 0.65 | 75.22 | 10.63 | 75.33 | 12.31 | 76.13 | 11.31 | 75.83 | 9.29 |
| 2-back  false alarms | 5.33 | 3.45 | 3.66 | 2.81 | 1.44 | 0.16 | 5.25 | 3.72 | 4.08 | 3.79 | 3.83 | 2.85 | 4.91 | 2.38 |

*CWMS: Categorization Working Memory Span task*

*RT: reaction times*

As done for behavioral data, we preliminarily carried out separate between-group ANOVAs for each task on the pre-training (T1 session) also for electrophysiological data, to test for possible *a priori* differences. As can be seen in Tables S5-S8, no between-group differences were found in the pre-training (T1 session).

**Table S5.** Preliminary analysis of pre-training (T1 session) for the CWMS task: word RP amplitude (200-230 ms after stimulus onset) elicited by target stimuli in posterior ROIs.

| **Statistical analysis** |  |  |  | ***F*(1,28)** | ***p* value** |
| --- | --- | --- | --- | --- | --- |
| Group main effect |  |  |  | 1.975 | 0.175 |
| Laterality main effect |  |  |  | 0.007 | 0.934 |
| Group by Laterality interaction | |  |  | 0.298 | 0.589 |

**Table S6.** Preliminary analysis of pre-training (T1 session) for the CWMS task: word RP amplitude (200-230 ms after stimulus onset) elicited by w1 stimuli (control condition) in posterior ROIs.

| **Statistical analysis** |  |  |  | ***F*(1,28)** | ***p* value** |
| --- | --- | --- | --- | --- | --- |
| Group main effect |  |  |  | 1.385 | 0.249 |
| Laterality main effect |  |  |  | 1.411 | 0.245 |
| Group by Laterality interaction | |  |  | 1.173 | 0.288 |

**Table S7.** Preliminary analysis of pre-training (T1 session) for the *2*-back task: P300 (450-500 ms after stimulus onset) elicited by target/standard stimuli in posterior ROIs.

| **Statistical analysis** |  |  |  | ***F*(1,28)** | ***p* value** |
| --- | --- | --- | --- | --- | --- |
| Group main effect |  |  |  | 0.002 | 0.968 |
| Stimulus main effect |  |  |  | 4.440 | 0.044 |
| Laterality main effect |  |  |  | 11.369 | 0.002 |
| Group by Stimulus interaction | |  |  | 3.267 | 0.081 |
| Group by Laterality interaction | |  |  | 2.759 | 0.108 |
| Group by Stimulus by Laterality interaction | |  |  | 3.474 | 0.067 |

**Table S8.** Preliminary analysis of pre-training (T1 session) for the sentence reading task: N400 amplitude (450-550 ms after stimulus onset) elicited by incongruent minus congruent stimuli in frontal and posterior ROIs.

| **Statistical analysis** |  |  |  | ***F*(1,28)** | ***p* value** |
| --- | --- | --- | --- | --- | --- |
| Group main effect |  |  |  | 0.042 | 0.838 |
| Region |  |  |  | 8.278 | 0.007 |
| Laterality main effect |  |  |  | 14.705 | < 0.001 |
| Group by Region interaction | |  |  | 0.184 | 0.671 |
| Group by Laterality interaction | |  |  | 1.522 | 0.228 |
| Group by Region by Laterality interaction | |  |  | 0.046 | 0.832 |
